# Transcranial Direct Current Stimulation Targeting the Entire Motor Network Does Not Increase Corticospinal Excitability

**DOI:** 10.3389/fnhum.2022.842954

**Published:** 2022-05-04

**Authors:** Joris Van der Cruijsen, Zeb D. Jonker, Eleni-Rosalina Andrinopoulou, Jessica E. Wijngaarden, Ditte A. Tangkau, Joke H. M. Tulen, Maarten A. Frens, Gerard M. Ribbers, Ruud W. Selles

**Affiliations:** ^1^Department of Rehabilitation Medicine, Erasmus MC, University Medical Center Rotterdam, Rotterdam, Netherlands; ^2^Department of Biomechanical Engineering, Delft University of Technology, Delft, Netherlands; ^3^Department of Neuroscience, Erasmus MC, University Medical Center Rotterdam, Rotterdam, Netherlands; ^4^Rijndam Rehabilitation Centre, Rotterdam, Netherlands; ^5^Department of Biostatistics, Erasmus MC, University Medical Center Rotterdam, Rotterdam, Netherlands; ^6^Department of Epidemiology, Erasmus MC, University Medical Center Rotterdam, Rotterdam, Netherlands; ^7^Department of Psychiatry, Erasmus MC, University Medical Center Rotterdam, Rotterdam, Netherlands; ^8^Department of Plastic and Reconstructive Surgery and Hand Surgery, Erasmus MC, University Medical Center Rotterdam, Rotterdam, Netherlands

**Keywords:** tDCS, TMS, corticospinal excitability, motor network, motor evoked potential

## Abstract

Transcranial direct current stimulation (tDCS) over the contralateral primary motor cortex of the target muscle (conventional tDCS) has been described to enhance corticospinal excitability, as measured with transcranial magnetic stimulation. Recently, tDCS targeting the brain regions functionally connected to the contralateral primary motor cortex (motor network tDCS) was reported to enhance corticospinal excitability more than conventional tDCS. We compared the effects of motor network tDCS, 2 mA conventional tDCS, and sham tDCS on corticospinal excitability in 21 healthy participants in a randomized, single-blind within-subject study design. We applied tDCS for 12 min and measured corticospinal excitability with TMS before tDCS and at 0, 15, 30, 45, and 60 min after tDCS. Statistical analysis showed that neither motor network tDCS nor conventional tDCS significantly increased corticospinal excitability relative to sham stimulation. Furthermore, the results did not provide evidence for superiority of motor network tDCS over conventional tDCS. Motor network tDCS seems equally susceptible to the sources of intersubject and intrasubject variability previously observed in response to conventional tDCS.

## Introduction

Research involving transcranial direct current stimulation (tDCS) has been growing exponentially since Nitsche and Paulus (2000) described its enhancing effects on the excitability of the motor system. Nitsche and Paulus (2000) applied transcranial magnetic stimulation (TMS) to assess changes in corticospinal excitability (CSE), reflected by motor evoked potentials (MEPs). They reported that motor evoked potentials significantly increased after 10 min of tDCS to the contralateral primary motor cortex (cM1). In tDCS motor studies, it has frequently been suggested that tDCS could lead to better motor learning (Antal et al., 2004; Reis et al., 2009; Stagg et al., 2011; Saucedo Marquez et al., 2013) and could benefit motor rehabilitation, for example after stroke (Hummel and Cohen, 2006; Santos Ferreira et al., 2019). On the other hand, however, other studies have failed to demonstrate a consistent effect of tDCS on corticospinal excitability (Bastani and Jaberzadeh, 2013; Wiethoff et al., 2014; Dyke et al., 2016; Jonker et al., 2021) and motor learning (Ammann et al., 2016; van der Vliet et al., 2017).

A significant part of the tDCS effectiveness research focuses on finding optimal stimulation parameters to improve the reliability and magnitude of tDCS effects. These stimulation parameters include stimulation duration (Nitsche and Paulus, 2000; Agboada et al., 2019), focality (Dmochowski et al., 2011), and location. For instance, stimulation of the premotor cortex, instead of the M1, has been found to result in a more robust increase in M1 excitability (Boros et al., 2008; Lefebvre et al., 2019). These findings indicate that stimulating other motor-related brain regions than the M1 can also modulate corticospinal excitability.

Recently, applying tDCS to regions functionally connected to the M1 was found to increase corticospinal excitability more than stimulation of M1 alone (Fischer et al., 2017). The rationale behind motor network tDCS was that the contralateral M1 does not act in isolation but communicates with functionally connected brain regions; consequently, brain regions connected to contralateral M1 influence the effect of stimulation on the contralateral M1. Therefore, Fischer et al. (2017) hypothesized that multifocal stimulation of the entire motor system would result in a larger change in corticospinal excitability. Although the stimulation field strength directly on the contralateral M1 was lower during motor network tDCS than for conventional tDCS, Fischer et al. (2017) the increase in corticospinal excitability was larger during motor network than conventional stimulation. Therefore, motor network tDCS may provide new leads to more effective tDCS interventions and a better understanding of the physiological basis of corticospinal excitability.

The promising results of motor network tDCS on corticospinal excitability have been described by only a single study. Since reproducibility in tDCS has been challenged due to low sample sizes (Minarik et al., 2016) and intersubject and intrasubject variability (Horvath et al., 2014, 2015; López-Alonso et al., 2014, 2015; Dyke et al., 2016; Jonker et al., 2021), replicating these findings is necessary to assess the reliability of motor network tDCS. Therefore, the primary goal of our study was to verify in a within-subject design if tDCS applied to the entire motor network leads to higher increases in corticospinal excitability than conventional tDCS targeting only the contralateral M1. The secondary goal of the study was to assess whether motor network tDCS and conventional tDCS increased corticospinal excitability compared to sham stimulation.

## Materials and Methods

### Participants

Twenty-one healthy subjects participated in this study (age: 18–30 years; 13 female). All participants gave written informed consent before the experiment. Participants were self-reported right-handed and free of known neuromuscular disorders. The study was approved by the Medical Ethics Review Board of the Erasmus University Medical Center (NL64529.078.18). All experimental procedures were conducted in accordance with the Declaration of Helsinki (2013).

### Experimental Design

#### Transcranial Direct Current Stimulation Conditions

Participants received non-invasive brain stimulation in three different tDCS configurations in a randomized, counterbalanced order in three experimental sessions separated by at least 48 h (Alonzo et al., 2012). Randomization of the applied stimulation configuration was performed *a priori* for the entire study. The participants were fully blinded and the investigators were partially blinded to the applied tDCS condition due to the different electrode locations in which stimulation electrodes were inserted for each stimulation configuration. To blind participants, stimulation electrodes were inserted in all nine electrode locations used in this experiment, regardless of whether the electrodes were used in a specific electrode configuration. Since the StarStim 8 only allows connecting eight electrodes, the investigators could not be blinded to the difference between motor network and conventional tDCS. However, investigators were blinded to the difference between motor network and sham tDCS.

All tDCS was applied using a StarStim 8 stimulator (Neuroelectrics, Barcelona, Spain) and a 128-channel EEG cap (TMSi, Oldenzaal, Netherlands) which was aligned according to the international 10/5 system (Oostenveld and Praamstra, 2001). We used platinum stimulation electrodes that could be manually inserted into any electrode location of the EEG cap. The surface contact area of the stimulation electrodes with the scalp was 0.79 cm^2^. We injected Sigma Gel (Parker Laboratories, Inc., Fairfield, NJ, United States) and used NIC 2.1 software (Neuroelectrics, Barcelona, Spain) to reduce the skin-electrode impedance below 2 kΩ when stimulation was applied.

##### Motor Network Transcranial Direct Current Stimulation

**FIGURE 1:**
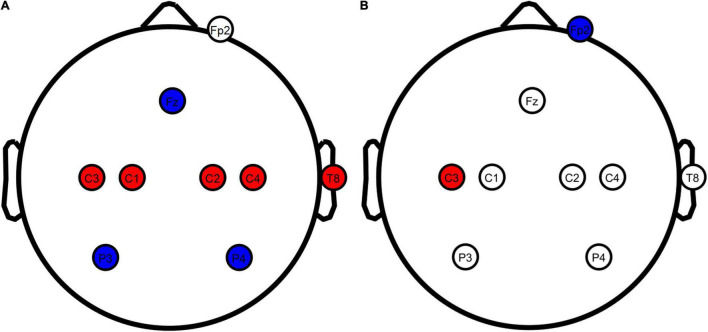
TDCS electrode configurations overview. **(A)** Motor network and sham tDCS configurations. **(B)** Conventional tDCS configuration. Red, anodes; blue, cathodes; white, electrodes not actively used for the stimulation configuration. Stimulation electrodes were inserted at all locations in all conditions to blind the participants from the applied configuration. Figure created using eeglab.

Motor network tDCS (Figure 1A) was performed as described by Fischer et al. (2017) to stimulate the entire motor network with 8 electrodes in total. Positive stimulation electrodes were placed over the primary motor cortices at C1, C2, C3, C4, T8 with input currents of 0.872, 0.888, 1.135, 0.922, and 0.183 mA, respectively. Negative stimulation electrodes were inserted at Fz, P3, and P4 with currents of −1.843, −1.121, and −1.035 mA, respectively. An additional electrode was inserted at the Fp2 channel (only actively used during conventional tDCS) to blind participants to the difference between motor network and conventional tDCS.

##### Conventional Transcranial Direct Current Stimulation

Conventional tDCS (Figure 1B) was based on Nitsche and Paulus (2000), with a single positive electrode placed over the contralateral primary motor cortex and a negative electrode on the ipsilateral supraorbital. Contrary to the original report by Nitsche and Paulus (2000), we placed the anode at C3 instead of directly above the motor hand area. C3 has been used as a standardized alternative in studies in which stimulation was applied through an EEG head cap, leading to similar changes in corticospinal excitability (Murray et al., 2015; Fischer et al., 2017; Rawji et al., 2018). Using standardized locations, we inserted a positive stimulation electrode at C3 and a negative stimulation electrode at Fp2. A 2-mA current was generated in between these electrodes to stimulate the contralateral primary motor cortex. Compared to motor network tDCS, the injection currents used for conventional tDCS lead to the highest current density (25.46 A/m^2^) at the scalp. This current density has been described as safe, with minimal sensation and no skin damage (Bikson et al., 2009). To blind the participants, stimulation electrodes were also inserted at the electrode locations used for motor network tDCS.

##### Motor Network Sham

Sham tDCS (Figure 1A) has been widely used as a control condition in tDCS/TMS research and mimics the sensation of active tDCS (Woods et al., 2016). Sham protocols only inject current at the beginning and the end of stimulation, resembling what participants experience in active stimulation conditions. We used the same electrode locations as the motor network condition in our sham stimulation.

#### Stimulation Protocol

The total stimulation duration was 12 min for all stimulation conditions. Both non-sham stimulation conditions consisted of three phases: (1) ramp up, in which the stimulation intensity linearly builds up from 0 to 100% in 60 s, (2) 10 min of constant stimulation at 100%, and (3) 60 s ramp down in which current linearly reduced from 100 to 0%. The sham condition was designed to give the same sensation as during active stimulation (Woods et al., 2016). It, therefore, consisted of a similar ramp-up phase of 60 s, directly followed by a ramp down phase of 60 s, which was repeated 10 min after the start of the stimulation session, resulting in a total duration of 12 min. In the sham condition, injection currents were built up to the same levels as motor network stimulation.

#### Corticospinal Excitability Measurements

We assessed corticospinal excitability (Barker et al., 1985) before we applied tDCS and at 0, 15, 30, 45, and 60 min after tDCS finished. Corticospinal excitability was assessed by measuring MEPs from the first dorsal interosseous (FDI) resulting from monophasic TMS pulses (MagPro X100 stimulator with an MC-B70 figure-eight coil, MagVenture, Farum, Denmark) applied to the motor hand area guided by a neuronavigational system (Polaris Spectra motion tracking system, NDI, Canada and visor2 software, ANT Neuro, Hengelo, Netherlands) to ensure MEP stability (Julkunen et al., 2009). We calculated MEPs from EMG activity, recorded at 5000 Hz with Ag/AgCl electrodes in a belly-tendon montage connected to a custom biosignal amplifier (TMSi, Oldenzaal, Netherlands).

From the EMG data, MEPs were online calculated as the largest peak-to-peak amplitude within 50 ms after a TMS pulse. The motor hand area was identified as the scalp location corresponding to the highest recorded MEPs. We stimulated at 50% of the maximum stimulator output on the motor cortex region (around C3 electrode) as an initial starting location to find the motor hand area. Throughout this process, we held the TMS coil tangent to the scalp, with the coil handle in the posterolateral direction rotated 45° from the midline. We increased the stimulation intensity in 5% increments until a scalp location was found for which the MEP exceeded 50 μV. At this location, about 10–20 pulses were required to determine the RMT, i.e., the stimulation intensity resulting in an MEP greater than 50 μV with a probability of 50% (Awiszus and Borckardt).

At this motor hand area, corticospinal excitability was assessed before tDCS (baseline) and 0, 15, 30, 45, and 60 min after tDCS using a fixed series of 65 TMS pulses on each time point. In these series, the inter-stimulus interval varied randomly between 2 and 5 s at a stimulation intensity of 120% of the RMT. Coil position and orientation relative to the scalp were monitored in real-time using the neuronavigational system to ensure a constant position throughout the measurement. EMG activity preceding the TMS pulses was also visually monitored. If persistent EMG activity was detected, we paused the TMS pulses and instructed participants to relax their muscles while providing real-time visual feedback on their EMG activity. The coil position during all TMS pulses and all EMG data were stored for offline analysis.

### Power Estimation

We estimated the statistical power to find a significant tDCS effect based on the MEP data published by Fischer et al. (2017). We considered the baseline-normalized data and identified the mean and standard errors of the mean (SEM) at time points directly after tDCS, and at 15, 30, and 60 min after the intervention. Due to the unbalanced distribution of these time points, the grand average of corticospinal excitability is biased toward early time points, where post-tDCS corticospinal excitability is generally lower. Therefore, we added a measurement point at 45 min after tDCS by linearly interpolating the MEP means and SEMs at 30 and 60 min to compensate for this bias. We calculated the statistical power to find a significant effect between motor network tDCS and conventional tDCS, between motor network tDCS and sham tDCS, and conventional tDCS and sham tDCS.

The power analysis was performed by simulation, assuming corticospinal excitability was normally distributed around each time point. For motor network tDCS and conventional tDCS, we considered the data of the left hemisphere, and for sham tDCS, we used the data recorded from the right M1 during conventional tDCS targeting the left M1. We converted all SEMs to standard deviations by multiplying with the square root of the sample size (15) of Fischer et al. (2017). Using the means and standard deviations, we calculated the MEP ratios (±SD) averaged over all time points (motor network tDCS: 1.324 ± 0.284; conventional tDCS: 1.151 ± 0.144; sham tDCS: 1.008 ± 0.151), removing the time information to enhance statistical power. We simulated 10,000 data sets for sample sizes ranging from 10 to 50 subjects. We applied a linear mixed effect model (see section “Analysis”) with a fixed intercept and fixed term for stimulation condition to each dataset to investigate if post-tDCS/pre-tDCS MEP ratio differed between tDCS configurations. For each sample size, we extracted the number of *p*-values below 0.05 as a measure for statistical power. The simulations showed that a sample size of 21 subjects had a power of 70% to find that motor network tDCS significantly (*p* < 0.05) increases the post/pre-tDCS MEP ratio compared to conventional stimulation. Additional computations showed that the inclusion of 21 subjects in a within-subjects design provided a power of 99% to find that motor network tDCS increases the MEP ratio compared to sham tDCS, and a power of 86% to find that conventional tDCS increases the MEP ratio compared to sham tDCS.

### Analysis

We calculated MEPs from the raw, continuous EMG data using EEGLAB (Delorme and Makeig, 2004) for experimental sessions. We first high-pass filtered the data (3 Hz, order: 1650) and then calculated MEPs as the peak-to-peak amplitude of the EMG signal within 50 ms after each TMS pulse. The mean and standard deviation (SD) of the coil position for all MEPs were calculated per session. Any TMS pulses applied while the coil position exceeded the mean coil position +3 SD were discarded from the analysis. Furthermore, TMS pulses in which the pre-TMS EMG amplitude in the 100 ms before the pulse exceeded the mean EMG amplitude +3 SD of all pulses within an experimental session were removed. The remaining pulses were considered the cleaned MEP data.

As a first step in the statistical analysis, we investigated if baseline corticospinal excitability differed between experimental sessions by applying a linear mixed-effects model. We defined two mixed-effects models with random intercepts per subject. For the full model, we included an intercept and the variable *stimulation condition* in the fixed-effects part. In both models, visual inspection indicated that baseline corticospinal excitability required a log-transform to ensure the residuals were normally distributed and homoscedastic. The likelihood ratio test was applied between both models to determine if baseline corticospinal excitability systematically varied between stimulation conditions. By doing so, we could identify potential systematic differences in baseline excitability that could intervene with any condition effects.

Since the variability in tDCS response has previously been attributed to intersubject variability, we screened for the presence of consistent responders in our sample. We looked for consistent responders in our data and defined those as subjects in which both network and conventional stimulation resulted in MEP ratios greater than one and were higher than the MEP ratio recorded during sham tDCS. We calculated the individual response to each stimulation condition as the ratio between the grand average of post-tDCS MEPs and baseline corticospinal excitability. As such, ratios above 1 correspond to enhanced cortical excitability, considered positive responses.

Next, we assessed the group effects of the different tDCS configurations on MEP ratios by applying a linear mixed-effects model with a random intercept per subject and a fixed effect term for *stimulation condition*. We evaluated two versions of the linear mixed-effects model. In the first model, visual inspection indicated log-transform was required for the outcome to ensure the residuals were both normally distributed and homoscedastic. In the second model, we performed a sensitivity analysis in which we removed outliers from the data, after which the residuals were normally distributed and homoscedastic, and the data was modeled accordingly. In this second mixed-effect model, we subtracted 1 from all MEP ratios, such that the intercept of the model corresponding to the average MEP ratio of sham stimulation could be interpreted. Finally, we investigated if the MEP ratio depended on baseline excitability by calculating the correlation coefficient between baseline excitability and the MEP ratio. After visually evaluating the distribution of MEP ratio and baseline corticospinal excitability, we calculated Pearson’s correlation coefficient for each stimulation condition. All statistical analyses were performed in MATLAB (MathWorks, Natick, MA, United States).

## Results

### Transcranial Magnetic Stimulation Data

Subject-specific resting motor threshold and baseline corticospinal excitability per condition are described in Supplementary Table 1. One participant (319) was unable to undergo the conventional stimulation protocol due to technical issues with the tDCS device. Within subjects, the mean (±SD) difference between the highest and lowest resting motor threshold (RMT) in all experimental sessions was 5% ± 3, indicating that RMTs were relatively constant over multiple sessions. On average, we discarded 6.2 ± 3.8 trials per subject from the analysis because the coil position deviated too much from the mean coil position or because too strong EMG activity preceded the TMS pulse. Comparison of the full and null linear mixed-effects models indicated the differences in baseline corticospinal excitability between sham stimulation (2203 ± 1562 μV), conventional tDCS (1753 ± 1349 μV), and motor network tDCS (2043 ± 1252 μV) were non-significant [*λ_*LR*_*(2) = 1.45, *p* = 0.485].

### Corticospinal Excitability – Subject Level

**FIGURE 2:**
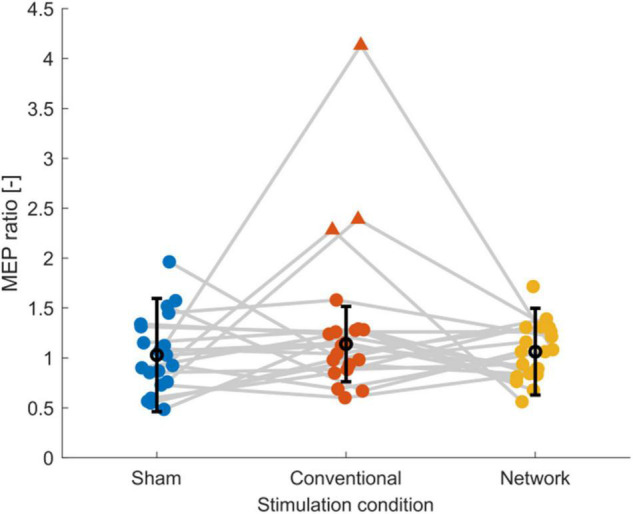
Scatter plot of the post-tDCS/pre-tDCS MEP ratio for sham, conventional, and network tDCS. Each data point corresponds to a single subject. Three subjects, shown as triangles, were considered outliers [outside the median (black marker) ± 1.5 times the interquartile range (error bars)], explaining the high standard deviations and the relatively high group response observed after conventional tDCS. Data of the same subject are connected with gray lines. Jitter was applied to the plot to enhance the readability. Figure created using eeglab.

Inspection of the MEP ratios for the different tDCS conditions (Figure 2) indicated that during conventional tDCS, three subjects were outliers compared to the rest of the subjects. These subjects had MEP ratios higher than 2, compared to the condition median of 1.01. The baseline excitability of these subjects was lower than the condition average (1860 ± 1297 μV) with 482.6, 489.6, and 514.1 μV. In our data, MEP ratios above 1 were found 11 times during sham, 12 times during conventional, and 11 times during network stimulation, but corticospinal excitability was not consistently modulated within subjects as hypothesized. More specifically, only one subject could be considered a consistent tDCS responder, i.e., showing an increase in corticospinal excitability for network and conventional tDCS greater than registered during sham stimulation. All other subjects had at least once MEP ratios below 1 for conventional or network tDCS or a stronger response from sham stimulation compared to conventional or network tDCS.

### Corticospinal Excitability – Group Level

The group-level statistical analysis on the post-tDCS/pre-tDCS MEP ratios revealed no effect for conventional stimulation [*b* = 0.198, *t*(59) = 1.755, *p* = 0.084] or network stimulation [*b* = 0.035, *t*(59) = 0.317, *p* = 0.753], indicating that averaged over all subjects tDCS did not enhance corticospinal excitability relative to sham stimulation. The average time courses of corticospinal excitability are shown in Supplementary Figure 1. Due to the three outliers identified in Figure 2 in the conventional tDCS response, we performed a sensitivity analysis to determine the effect of outliers on the statistical analysis. When we excluded the three outlier subjects from all three conditions and applied the linear mixed-effects model, the conventional condition effect [MEP ratio (mean ± SD): 1.05 ± 0.26 μV] diminished [*b* = −0.005, *t*(50) = −0.052, *p* = 0.959] and the network effect [MEP ratio (mean ± SD): 1.05 ± 0.26 μV] remained equally low [*b* = −0.007, *t*(50) = −0.071, *p* = 0.944]. In addition, the model’s intercept [*b* = 0.061, *t*(50) = 0.822, *p* = 0.415], i.e., the response to sham tDCS [MEP ratio (mean ± SD): 1.06 ± 0.40 μV], indicated that there was no mean change in corticospinal excitability for the control condition. Overall, these results indicate that MEP ratios were highly variable and on average for both conventional and motor network tDCS were equal to sham stimulation. Finally, we found that baseline excitability did not correlate with the MEP ratio of sham (*p* = 0.27), conventional (*p* = 0.05), or network tDCS (*p* = 0.14).

## Discussion

The primary goal of this study was to investigate if tDCS targeting the motor network of healthy subjects leads to larger changes in corticospinal excitability compared to conventional stimulation. Compared to sham stimulation, our sample of 21 healthy participants showed no significant increase in corticospinal excitability after motor network tDCS or conventional tDCS. Consequently, the results did not provide evidence for the superiority of motor network tDCS over conventional tDCS.

Our study is, to our knowledge, the first that attempts to replicate the effect of motor network tDCS on corticospinal excitability. We conducted the experiment in a larger sample (*n* = 21) than the original study (*n* = 15) (Fischer et al., 2017). Furthermore, we added sham stimulation as a control condition to distinguish potential TMS effects from tDCS effects. Nonetheless, no effect of motor network tDCS relative to conventional or even sham stimulation was found in our study. The inability to replicate the effects of motor network tDCS on corticospinal excitability fits with previous studies that recently challenged the potential of conventional tDCS (Wiethoff et al., 2014; Horvath et al., 2016; Tremblay et al., 2016; Jonker et al., 2021) and hd-tDCS (Pellegrini et al., 2020) to enhance corticospinal excitability.

Several factors may explain why we found no effect of motor network tDCS on corticospinal excitability. First, we did not control for intersubject and intrasubject variability in baseline corticospinal excitability. Previous studies that described positive effects of anodal tDCS controlled baseline corticospinal excitability by adjusting TMS intensity to elicit MEPs between 1 and 1.5 mV (Nitsche et al., 2004; Kuo et al., 2013). However, this would increase the intersubject variability in stimulation intensity, increasing the likelihood of stimulation intensity being an extraneous variable. Importantly, we found baseline corticospinal excitability did not correlate with the change in corticospinal excitability after tDCS. Also, baseline corticospinal excitability did not significantly vary between sessions in our sample. Furthermore, several studies show that adjusting TMS intensity to control for baseline corticospinal excitability is not a prerequisite for finding positive tDCS effects (Lang et al., 2004; Nitsche et al., 2004; Di Lazzaro et al., 2012; Pellicciari et al., 2013). Therefore, we do not consider variability in baseline excitability to explain the absence of a tDCS effect in our study.

A second factor explaining why tDCS did not affect corticospinal excitability could be the applied TMS protocol. In our study, the number of TMS pulses was relatively high (65 per interval; 390 per session) and the inter-stimulus-interval relatively short (2–5 s), which can affect corticospinal excitability (Julkunen et al., 2012; Pellicciari et al., 2016), and potentially intervene with a tDCS effect. However, a similar number of pulses has been used to demonstrate the enhancing effect of anodal tDCS on corticospinal excitability (Wiethoff et al., 2014). Furthermore, we used a sham condition to distinguish tDCS from potential effects on corticospinal excitability introduced by the TMS protocol. Our statistical analysis revealed no effect of sham stimulation on corticospinal excitability. Consequently, we argue that our TMS protocol did not interfere with potential tDCS effects (Horvath et al., 2015). We also inspected the response per subject for the three stimulation conditions to investigate subgroups of tDCS *responders* in our sample. Previous research has shown that in the absence of group effects, subgroups of tDCS responders may exist (Wiethoff et al., 2014). While MEP ratios above 1 were found in all conditions, there was no consistent corticospinal excitability enhancement for conventional and network tDCS in individual subjects. Only in one subject, conventional and network tDCS resulted in a stronger increase of cortical excitability compared to sham stimulation.

Finally, we applied TMS only on the contralateral motor cortex, which is different compared to Fischer et al. (2017), who applied TMS to both hemispheres. Thus, the effect of motor network tDCS described by Fischer et al. (2017) could originate from a combination of bilateral mixed TMS and tDCS and therefore be absent in our current study. An additional difference is the smaller electrode size we used for tDCS. Smaller electrodes lead to more focal electric fields in the brain (Mikkonen et al., 2020). Together with the standardized electrode locations we used, it could thus be that the peak electric fields were not located at the intended M1 target due to interindividual differences in brain anatomy relative to standardized EEG locations (Scrivener and Reader, 2022). Nonetheless, modeling studies indicate that the small stimulation electrodes generate electric fields in M1 that exceed those of large electrodes (up to 35 cm^2^) in a broad cortical area (Mikkonen et al., 2020), supporting the use of small electrodes in standardized EEG locations.

There are some limitations in our study that need to be considered. First, the primary goal of this study is somewhat limited by the relatively low power for the comparison between motor network tDCS and conventional tDCS. Our *a priori* power calculation, based on the data published by Fischer et al. (2017), indicated that our within-subject design of 21 healthy participants provided 70% chance of finding an effect of motor network tDCS relative to conventional tDCS. Thus, we should be careful with concluding that motor network stimulation is non-superior compared to conventional stimulation. However, because we did not find effects of both motor network tDCS and conventional tDCS relative to sham tDCS, despite high *a priori* powers for these comparisons (99 and 86%, respectively), it is unlikely that one intervention worked better than the other.

An additional limitation follows from the conventional sham protocol, which is under debate because it was shown that sham stimulation could not reliably mask active stimulation from sham stimulation in within-subject design studies (Ambrus et al., 2012; Fonteneau et al., 2019; Greinacher et al., 2019). Recently, new protocols have been suggested to better blind participants from active stimulation, for instance, by continuous stimulation in a montage that exceeds the skin’s perception threshold but is not strong enough to pass the skull (Neri et al., 2020). However, we were unaware of this alternative type of sham stimulation at the start of the data collection of this study. Although these limitations with the used sham protocol exist, it allowed us to distinguish potential effects on corticospinal excitability of the used TMS protocol from the potential effects of tDCS. However, questionnaires about the participants’ awareness of the used tDCS configurations could have helped control awareness-related tDCS response effects.

Finally, our statistical analysis did not control for sources of intersubject variability, such as genetics (Antal et al., 2010; Teo et al., 2014; van der Vliet et al., 2017), the electric field strength at stimulated brain areas (Laakso et al., 2015, 2019), or intrasubject variability, such as circadian or hormonal cycles (Horvath et al., 2014). One source of intrasubject variability was caused by the difference in stimulation currents of motor network tDCS and conventional tDCS, which resulted in different current densities at the contralateral M1. Due to safety constraints (Bikson et al., 2009), it was not possible to match the current densities between the two conditions. Consequently, it remains an open question whether the original findings of motor network tDCS are due to stimulation of the entire motor network or if they reflect the previously described non-linear relationship between the tDCS response and electric field strength at the contralateral M1 (Batsikadze et al., 2013).

## Conclusion

Our study provides no evidence that motor network tDCS or conventional tDCS increases corticospinal excitability compared to sham tDCS. Consequently, the results did not provide evidence for superiority of motor network tDCS over conventional tDCS. While the rationale for tDCS targeting the entire motor network could be valid from the neurophysiological perspective, our results indicate that motor network tDCS might be equally susceptible to sources of intrasubject and intersubject variability as previously demonstrated for conventional tDCS. Including neurophysiologic measures such as EEG or magnetic resonance spectroscopy to control intrasubject and intersubject variability may facilitate the exploration of the potential of motor network tDCS and tDCS in general.

## Data Availability Statement

The raw data supporting the conclusions of this article will be made available by the authors, without undue reservation.

## Ethics Statement

The studies involving human participants were reviewed and approved by the Medical Ethics Review Board of the Erasmus University Medical Center (NL64529.078.18). The patients/participants provided their written informed consent to participate in this study.

## Author Contributions

JV and ZJ were involved in the conceptualization of the study. JV, ZJ, and E-RA performed the formal analysis. JV, JW, and DT collected the data. JT and RS provided resources to perform the experiment. MF, GR, and RS supervised the study. JV wrote the original draft. All authors reviewed the manuscript.

## Conflict of Interest

The authors declare that the research was conducted in the absence of any commercial or financial relationships that could be construed as a potential conflict of interest.

## Publisher’s Note

All claims expressed in this article are solely those of the authors and do not necessarily represent those of their affiliated organizations, or those of the publisher, the editors and the reviewers. Any product that may be evaluated in this article, or claim that may be made by its manufacturer, is not guaranteed or endorsed by the publisher.
