# Supplementary material for: Transcranial Direct Current Stimulation Targeting the Entire Motor Network Does Not Increase Corticospinal Excitability
Source: Front Hum Neurosci. 2022 May 4;16:842954. doi: 10.3389/fnhum.2022.842954 (PMC9114302; doi:10.3389/fnhum.2022.842954)
Supplement: Supplementary file 1 [file Data_Sheet_1.docx]

# Supplementary material


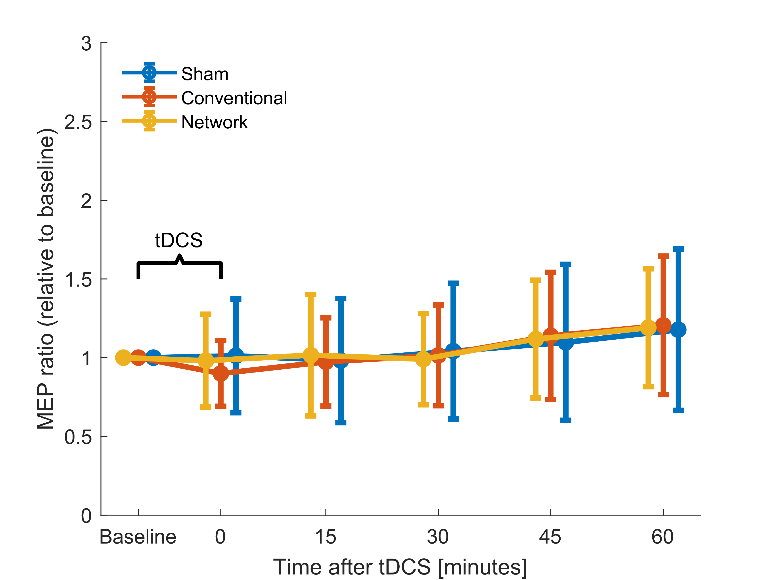


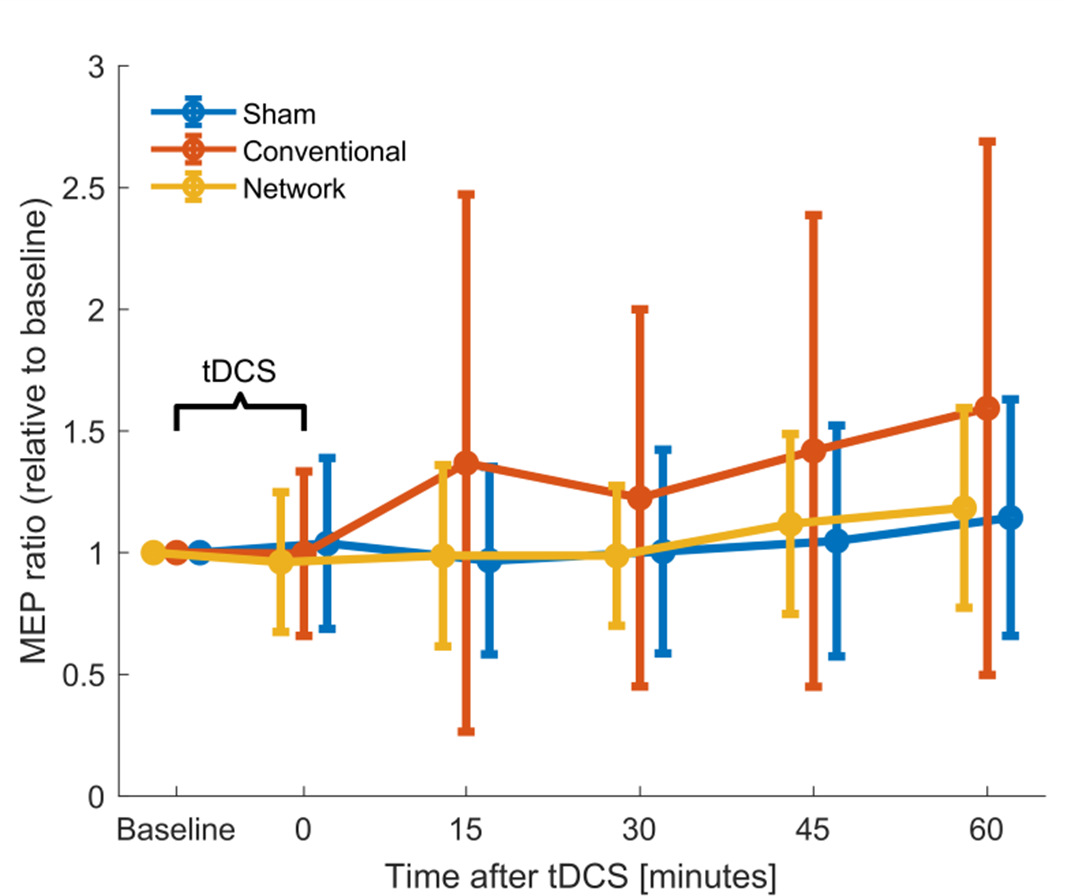


Supplementary Figure 1. Group average change in corticospinal excitability, averaged over all subjects. The x-axis shows time in minutes, and the y-axis the baseline normalised MEPs in µV. The error bars reflected standard deviations over the subjects per time point. Left: time courses of corticospinal excitability before the removal of 3 outlier subjects. Right: time courses of corticospinal excitability after the removal of 3 outlier subjects.

Supplementary Table 1. Baseline corticospinal excitability and resting motor threshold (RMT) per participant.

|  |  | **Sham** | |  | **Conventional** | |  | **Motor Network** | |
| --- | --- | --- | --- | --- | --- | --- | --- | --- | --- |
| **Subject** |  | **Baseline** | **RMT** |  | **Baseline** | **RMT** |  | **Baseline** | **RMT** |
| **301** |  | 2314.3 | 68 |  | 490.2 | 71 |  | 3680.3 | 71 |
| **302** |  | 660.4 | 51 |  | 489.6 | 48 |  | 3511.6 | 54 |
| **303** |  | 3089.4 | 63 |  | 1449.7 | 59 |  | 1076.7 | 56 |
| **304** |  | 1788.7 | 52 |  | 1651.4 | 51 |  | 2601.8 | 54 |
| **305** |  | 2248.9 | 53 |  | 3558.1 | 60 |  | 1436.1 | 52 |
| **306** |  | 3289.2 | 66 |  | 2360.2 | 60 |  | 1996.0 | 62 |
| **307** |  | 1450.1 | 68 |  | 6051.9 | 66 |  | 2282.1 | 65 |
| **308** |  | 497.7 | 68 |  | 921.0 | 75 |  | 605.6 | 76 |
| **309** |  | 908.1 | 71 |  | 1752.2 | 72 |  | 1096.8 | 70 |
| **310** |  | 2525.4 | 48 |  | 653.0 | 46 |  | 1210.7 | 52 |
| **311** |  | 1889.9 | 50 |  | 1991.8 | 51 |  | 2068.4 | 48 |
| **312** |  | 1261.5 | 54 |  | 1261.9 | 58 |  | 1232.6 | 62 |
| **313** |  | 3084.4 | 44 |  | 1185.1 | 44 |  | 2449.3 | 44 |
| **314** |  | 7500.7 | 40 |  | 3253.2 | 37 |  | 4026.1 | 40 |
| **315** |  | 2692.4 | 61 |  | 2635.9 | 59 |  | 2053.5 | 62 |
| **316** |  | 1000.3 | 59 |  | 1916.1 | 63 |  | 5514.4 | 56 |
| **317** |  | 1276.2 | 53 |  | 1461.8 | 55 |  | 1326.5 | 56 |
| **318** |  | 705.6 | 68 |  | 958.2 | 65 |  | 874.2 | 74 |
| **319** |  | 1458.0 | 63 |  | NA | NA |  | 1820.0 | 66 |
| **320** |  | 4325.6 | 59 |  | 2603.4 | 53 |  | 1286.0 | 62 |
| **321** |  | 2487.3 | 80 |  | 548.5 | 83 |  | 729.6 | 88 |

Baseline: corticospinal excitability (μV) averaged over all pre-tDCS MEPs; RMT: resting motor threshold in percentage of maximum stimulator output.
